# Abemaciclib early access - an Italian experience

**DOI:** 10.3389/fonc.2025.1563844

**Published:** 2025-09-29

**Authors:** Concetta Calabrò, Eleonora Cannella, Patrizia Nardulli

**Affiliations:** 1 Struttura Complessa di Farmacia IRCCS Istituto Tumori “Giovanni Paolo II”, Bari, Italy; 2 Pharmacy Department, Provincial Health Authority of Syracuse, Syracuse, Italy

**Keywords:** early access, cost - saving, breast cancer, C(nn) category, sustainability

## Abstract

Italian legislation allows pharmaceutical companies to market drugs authorized through the centralized procedure before price negotiation with the Italian Medicines Agency (AIFA), placing them in the C(nn) category, which indicates non-reimbursed and non-negotiated drugs. Since April 4, 2022, the manufacturer of Verzenios^®^ (Abemaciclib) has made the drug available at a nominal price under the C(nn) classification for its adjuvant treatment of high-risk breast cancer. The MonarchE study showed that Verzenios^®^, combined with endocrine therapy (ET), reduced the risk of recurrence and distant metastasis by 35% in high-risk patients. The treatment was continued for 24 months, and benefits, including invasive disease-free survival (IDFS) and distant relapse-free survival (DRFS), persisted for up to 48 months, confirming long-term efficacy and tolerability. Results indicated fewer breast cancer-related deaths in the Abemaciclib plus ET arm (4.2%) compared to the ET-only arm (4.9%). Additionally, patients in the control group developed metastatic disease at nearly twice the rate of those receiving Abemaciclib. In Italy, approximately 2,000 patients were treated with Verzenios^®^ in the first year under the C(nn) category without affecting the National Health System’s budget, due to the drug’s symbolic cost. This approach resulted in significant savings by avoiding the full cost of adjuvant therapy and preventing metastatic recurrences. Specifically, managing relapses costs €15,000 per patient, saving around €525,000 per 100 patients treated. Additional savings are expected in the third year, as patients will not require metastatic breast cancer treatment, saving around €17,000 per patient.

## Introduction

Italian legislation allows pharmaceutical companies to market a drug authorized through a centralized procedure prior to the conclusion of the Italian Medicines Agency’s (AIFA) price negotiation process, placing the drug in a specific classification for those not yet evaluated for reimbursement. This class is referred to as C(nn), where “C” denotes a class of drugs not reimbursable by the National Health System, and “(nn)” stands for “non-negotiated.

On April 4, 2022, following European Medicines Agency (EMA) authorization, Verzenios^®^ in combination with endocrine therapy, was approved for the adjuvant treatment of adult patients with early-stage breast cancer that is hormone receptor-positive (HR+), human epidermal growth factor receptor 2-negative (HER2-), lymph node-positive, and at high risk of recurrence (1). Verzenios^®^ thus became the first and only CDK 4/6 inhibitor authorized in the European Union for adjuvant therapy. In line with AIFA’s classification of Verzenios^®^ as C(nn), and to address an unmet therapeutic need while awaiting the completion of the standard reimbursement process for the new indication, the manufacturer of Abemaciclib made the drug available in the adjuvant therapy setting at a symbolic price.

More specifically, the symbolic cost was €1.00 per pack, regardless of the dosage. During the C(nn) phase, the cost of Abemaciclib is borne entirely by the individual healthcare facilities dispensing the drug. Since the price has not yet been negotiated with AIFA, there is no national reimbursement, and the expenditure directly impacts the hospital authority’s budget.

## Materials and methods

In Italy, recognizing the unmet therapeutic need addressed by the drug, the manufacturer decided to make Verzenios^®^ available in the adjuvant therapy indication under the C(nn) classification.

### Clinical requirements

#### Therapeutic need

##### Urgency

The therapeutic need is critical due to the lack of alternative treatment options for this specific indication.

##### Disease severity

Breast cancer is the leading cause of cancer-related deaths among women in Italy, with an estimated 12,300 deaths in 2020. Despite the efficacy of surgery and standard adjuvant therapies, up to 30% of patients with hormone receptor-positive (HR+), HER2-negative breast cancer will experience recurrence, often in the form of incurable distant metastases (2–9). The risk of recurrence is particularly high within the first two years following diagnosis. Additionally, breast cancer is the most commonly diagnosed malignancy among women, representing 1 in 3 female cancer cases (10–12).

#### Added therapeutic value

Verzenios^®^ is the first and only CDK 4/6 inhibitor with dual therapeutic indications, being approved for both metastatic breast cancer (MBC) and early breast cancer (EBC) (13–15). It has demonstrated efficacy and good tolerability in both settings and was evaluated in patients with particularly aggressive disease (16–20):

##### MBC

Verzenios^®^ was evaluated in the Monarch 2 and Monarch 3 trials, which uniquely targeted a homogeneous population of endocrine-resistant and endocrine-sensitive patients, making it the only CDK 4/6 inhibitor with such consistent study populations. In Monarch 2, Verzenios^®^ showed significant efficacy, meeting the primary endpoint of progression-free survival (PFS), with a 5-year PFS of 21.25 months compared to 5 months in the control arm. In Monarch 3, the primary endpoint of PFS at 5 years was 26.7 months versus 9.6 months in the control group (21–30).

Additionally, long-term follow-up data from Monarch 2 with 80 months of observation showed an overall survival (OS) estimate of 41.2 months versus 29.2 months in the control arm at 5 years. The OS data from Monarch 3, while not yet definitive, indicate a promising trend, with an OS estimate of 67.7 months versus 54.5 months in the control arm. Verzenios^®^ also demonstrated an excellent safety profile across both trials (31–36).

It is important to note that studies on other CDK 4/6 inhibitors available on the market have shown negative results for overall survival (OS) or have targeted a heterogeneous patient population, including both endocrine-resistant and endocrine-sensitive patients (ER-ES), unlike the homogeneous populations studied in the Monarch 2 and 3 trials (37–41).

##### EBC (approved indication)

In the MonarchE study, it was demonstrated that Verzenios^®^, in combination with endocrine therapy (ET), reduces the risk of recurrence and distant metastasis by 35% in patients with high-risk disease (≥4 positive axillary lymph nodes [ALN+] or 1–3 ALN+ with additional high-risk factors such as grade 3 tumors and/or tumor size ≥ 5 cm) (16–20). Patients received treatment for 24 months, the clinical benefits, including invasive disease-free survival (IDFS) and distant relapse-free survival (DRFS), were sustained at 48 months, with the drug being well tolerated (42–47).

Furthermore, although overall survival (OS) data are not yet available, fewer breast cancer-related deaths were observed in the Abemaciclib plus ET arm compared to the ET alone arm, with 117 deaths (4.2%) versus 138 deaths (4.9%). Additionally, nearly twice as many patients in the control arm developed and are currently living with metastatic disease compared to those who received Abemaciclib (48). Follow-up is ongoing until the final survival assessment.

### Clinical summary

The significant clinical outcomes in early breast cancer (EBC), with a 35% reduction in invasive disease-free survival (IDFS) and distant relapse-free survival (DRFS) achieved through 24 months of preventative treatment with endocrine therapy (ET) and Verzenios^®^, allow us to conclude the following (42–45):

1. Reduction in Metastatic Recurrence (MR): Among 100 patients treated with Verzenios® for 24 months, 35 will avoid metastatic recurrence, with the clinical benefit persisting for 48 months, as demonstrated by current follow-up data, which indicate a sustained effect beyond 48 months.
2. Long-term Disease Management: The treatment provides optimal management of the disease, improving patients’ quality of life and health outcomes over time.
3. Reduction in MBC Treatment Burden: Fewer patients will require treatment for metastatic breast cancer (MBC) as a result of this early intervention (10–12).

## Discussion

### Sustainability requirements for the national health system

In Italy, early breast cancer (EBC) in high-risk HR+/HER2- patients constitutes a relatively small population, comprising only 3,408 individuals, or approximately 6% of new EBC diagnoses across the entire population. Specifically, it is projected that 66.2% of the 55,000 new breast cancer diagnoses anticipated in 2022 will involve patients with HR+ and HER2- tumors, equating to about 36,410 cases. Considering that 90% of these patients are diagnosed with non-metastatic cancer, the estimated non-metastatic population is approximately 32,769 patients. By applying the 13% prevalence rate of high-risk patients in Italy to this non-metastatic cohort, we can hypothesize that around 4,260 patients may be eligible for treatment. Of these, 80% would be candidates for Abemaciclib, resulting in an estimated 3,408 patients—representing only 6% of the total breast cancer population (10–12).

The Italian experience with Verzenios^®^ involved approximately 2,000 patients over the course of one year, with no financial impact on the National Health System due to the symbolic pricing of the drug. This approach also resulted in additional savings 49:

#### Avoidance of full-price drug expenses

By using Verzenios^®^ for the management of adjuvant disease, there were significant savings from avoiding the cost of drugs that are not currently authorized for adjuvant use (50–52).

#### Savings on metastatic recurrences

Based on findings from real-world evidence (RWE) (53, 54), thexcosts avoided for managing metastatic recurrences are approximately €15,000 per patient. For every 100 patients treated, this translates to an estimated savings of about €525,000, assuming 35 patients remain free of metastatic recurrence (MR).

#### Long-term cost avoidance

From the third year onward, further costs are avoided due to the sustained clinical benefits lasting for 48 months, as these patients will not require treatment for metastatic breast cancer (MBC). The average cost for current therapies is approximately €17,000 per patient (55).

The introduction of Abemaciclib (Verzenios^®^) in Italy under the class C (nn) framework represents a significant case study in the management of early access to innovative therapies. This regulatory pathway allowed the drug to be made available to patients without awaiting the full pricing and reimbursement approval process by AIFA, thereby accelerating access for individuals with unmet medical needs, specifically in the adjuvant treatment of breast cancer.

### Regulatory, market, and clinical context

In Italy, the C(nn) classification provides a temporary pathway for drugs centrally authorized by the European Medicines Agency (EMA) but still under national pricing and reimbursement negotiations. By granting access at a symbolic or nominal price, it bridges the gap between regulatory approval and market availability, allowing patients with unmet medical needs to benefit from innovative therapies without delay.

The final negotiated price is typically much higher than the symbolic pre-negotiation cost. Although this difference could, in theory, discourage early access, many manufacturers are willing to supply the drug during the C(nn) phase under specific conditions. This early availability offers clinicians valuable opportunities to gain first-hand experience with the drug—assessing its real-world clinical effectiveness, safety profile, and management of adverse events—before large-scale adoption.

However, the C(nn) mechanism’s ability to expand access is inherently limited. Since net revenue for companies during this phase is essentially zero, there is little incentive to promote widespread use, and distribution generally occurs on a case-by-case basis following individual clinical requests.

Pricing policies also vary considerably. Not all manufacturers opt for a symbolic price; in some cases, drugs are offered at their full commercial price prior to negotiation, which can be prohibitively high. This variability strongly influences uptake: when no discount is applied, healthcare facilities may refrain from using the C(nn) pathway, especially in the absence of dedicated funding. Such inconsistencies risk undermining both equity and effectiveness.

Although the C(nn) classification clearly enables earlier availability of new medicines, its actual impact on access remains difficult to measure. AIFA’s OsMed reports provide only aggregated data and do not specifically track C(nn) utilization, and there is no publicly available dataset comparing use before and after price negotiation. This lack of transparency limits comprehensive assessment and highlights the need for systematic data collection on early access programs.

By offering Abemaciclib at a symbolic price, the manufacturer demonstrated its commitment to patient access while negotiations with AIFA were ongoing. This approach is particularly relevant in the context of adjuvant breast cancer treatment, where timely intervention is critical to prevent disease progression or recurrence. Early access to Abemaciclib allows patients to start their therapy without delays, potentially avoiding the high medical and social costs associated with metastatic recurrences.

From a clinical standpoint, the inclusion of Abemaciclib in class C(nn) helps close the therapeutic gap between regulatory authorization and real-world availability, equipping oncologists with an additional option for managing high-risk breast cancer patients in Italy. More broadly, this example reflects an evolving trend toward greater flexibility in drug access policies, especially in oncology, where timely intervention can decisively influence patient outcomes.

Furthermore, cost-effectiveness analyzes conducted in the United Kingdom, Canada, and the United States consistently suggest that Abemaciclib would require price reductions in the range of 80–90% to meet commonly accepted thresholds for reimbursement (56, 57). These findings highlight the economic challenge of introducing high-cost therapies and support the rationale for temporary early access mechanisms such as the C(nn) classification in Italy.

## Conclusion

The Italian experience with Abemaciclib in class C(nn) offers valuable insights into the potential benefits and challenges of early drug access frameworks.

This case highlights the importance of collaboration between regulatory agencies, pharmaceutical companies, and healthcare providers to ensure that early access mechanisms are used effectively and equitably. Additionally, the experience with Abemaciclib could inform future policies, potentially leading to refinements in the C(nn) classification system or the broader approach to early access and market entry for innovative drugs in Italy.

In conclusion, the early access to Abemaciclib through the C(nn) pathway represents a meaningful step toward balancing rapid drug availability with regulatory and economic considerations. As more innovative therapies are developed and enter the market, similar models may become increasingly important for ensuring timely access to life-saving treatments, particularly in oncology and other high-need therapeutic areas.

## Data Availability

Publicly available datasets were analyzed in this study. This data can be found here: MonarchE study https://cdn.clinicaltrials.gov/large-docs/97/NCT03155997/Prot_000.pdf.

## References

[1] Hortobagyi GN Connolly JL D’Orsi CJ Edge SB Byrd DR Compton CC . Chapter 48: breast. In: Edge SB Byrd DR Compton CC , editors. AJCC cancer staging manual, 8th edition. Springer, New York (2017). p. 771–89.

[2] Cardoso F Spence D Mertz S Corneliussen-James D Sabelko K Gralow J . Global analysis of advanced/metastatic breast cancer: Decade report (2005-2015). Breast. (2018) 39:131–8. doi: 10.1016/j.breast.2018.03.002, PMID: 29679849

[3] Cheng L Swartz MD Zhao H Kapadia AS Lai D Rowan PJ . Hazard of Recurrence among Women after Primary Breast Cancer Treatment—A 10-Year Follow-up Using Data from SEER-Medicare. Cancer Epidemiol Biomarkers amp; Prev. (2012) 21:800. doi: 10.1158/1055-9965.EPI-11-1089, PMID: 22426147

[4] Pan H Gray R Braybrooke J Davies C Taylor C McGale P . 20-Year Risks of Breast-Cancer Recurrence after Stopping Endocrine Therapy at 5 Years. N Engl J Med. (2017) 377:1836–46. doi: 10.1056/NEJMoa1701830, PMID: 29117498 PMC5734609

[5] Colleoni M Sun Z Price KN Karlsson P Forbes JF Thürlimann B . Annual hazard rates of recurrence for breast cancer during 24 years of follow-up: results from the international breast cancer study group trials I to V. J ClinOncol. (2016) 34:927–35. doi: 10.1200/JCO.2015.62.3504, PMID: 26786933 PMC4933127

[6] Cardoso F Paluch-Shimon S Senkus E Curigliano G Aapro MS André F . 5th ESO-ESMO international consensus guidelines for advanced breast cancer (ABC 5). Ann Oncol. (2020) 31:1623–49. doi: 10.1016/j.annonc.2020.09.010, PMID: 32979513 PMC7510449

[7] Christiansen P Al-Suliman N Bjerre K . Recurrence pattern and prognosis in low-risk breast cancer patients – Data from the DBCG 89-A programme. Acta Oncologica. (2008) 47:691–703., PMID: 18465337 10.1080/02841860802056594

[8] Marchetti P . Tumore del seno: più guarigioni con la terapia estesa dopo la chirurgia (dottnet.it) . Available online at: https://www.dottnet.it/articolo/32529211/tumore-del-seno-piu-guarigioni-con-la-terapia-estesa-dopo-la-chirurgia-/ (Accessed 20 December 2021).

[9] Davies C Pan H Godwin J Gray R Arriagada R Raina V Adjuvant Tamoxifen: Longer Against Shorter (ATLAS) Collaborative Group . Long-term effects of continuing adjuvant tamoxifen to 10 years versus stopping at 5 years after diagnosis of estrogen receptor-positive breast cancer: ATLAS, a randomized trial. . Lancet. (2013) 381:805–16. doi: 10.1016/S0140-6736(12)61963-1, PMID: 23219286 PMC3596060

[10] AIOM AIRTUM SIAPEC-IAP . I numeri del cancro in italia. (2020).

[11] Tagliabue G Fabiano S Contiero P Barigelletti G Castelli M Mazzoleni G . AIRTUM working group, molecular subtypes, metastatic pattern and patient age in breast cancer: an analysis of italian network of cancer registries (AIRTUM) data. J Clin Med. (2021) 10:5873. doi: 10.3390/jcm10245873, PMID: 34945169 PMC8706111

[12] Slamon DJ Clark GM Wong SG Levin WJ Ullrich A McGuire WL . Human breast cancer: correlation of relapse and survival with amplification of the HER-2/neu oncogene. Science. (1987) 235:177–82., PMID: 3798106 10.1126/science.3798106

[13] AIOM AIRO A.N.I.S.C SIAPEC-IAP SICO, SIRM Linee guida neoplasie della mammella. Edizione. (2021)

[14] Eli Lilly & Company . Protocol I3Y-MC-JPCF. In: monarchE: A randomized, open-label, phase 3 study of abemaciclib combined with standard adjuvant endocrine therapy versus standard adjuvant endocrine therapy alone in patients with high risk, node positive, early stage, hormone receptor positive, human epidermal receptor 2 negative, breast cancer (2020).

[15] Brown J Method MW Nelson DR . Abstract P5-08-18: Mortality rates associated with clinical and pathological characteristics of hormone receptor positive human epidermal growth factor receptor 2 negative early breast cancer: An analysis of the 2010–2016 surveillance, epidemiology, and end results data. Cancer Res. (2020) 80:P5–08-18-P5-08-18. doi: 10.1158/1538-7445.SABCS19-P5-08-18

[16] Patnaik A Rosen LS Tolaney SM Tolcher AW Goldman JW Gandhi L . Efficacy and safety of abemaciclib, an inhibitor of CDK4 and CDK6, for patients with breast cancer, non-small cell lung cancer, and other solid tumors. Cancer Discov. (2016) 6:740–53. doi: 10.1158/2159-8290.CD-16-0095, PMID: 27217383

[17] Beeram M Tolaney SM Beck JT Dickler MN Conlin AK Dees EC . A phase 1b study of abemaciclib, an inhibitor of CDK4 and CDK6, in combination with endocrine and HER2-targeted therapies for patients with metastatic breast cancer. Ann Oncol. 27:vi556.

[18] Tate SC Sykes AK Kulanthaivel P Chan EM Turner PK Cronier DM . A population pharmacokinetic and pharmacodynamic analysis of abemaciclib in a phase I clinical trial in cancerPatients. Clin Pharmacokinet. (2018) 57:335–44. doi: 10.1007/s40262-017-0559-8, PMID: 28540640 PMC5814509

[19] Dickler MN Tolaney SM Rugo HS Cortés J Diéras V Patt D . MONARCH 1, A phase II study of abemaciclib, a CDK4 and CDK6 inhibitor, as a single agent, in patients with refractory HR+/HER2- metastatic breast cancer. Clin Cancer Res. (2017) 23:5218–24. doi: 10.1158/1078-0432.CCR-17-0754, PMID: 28533223 PMC5581697

[20] Hurvitz SA Martin M Press MF Chan D Fernandez-Abad M Petru E . Potent Cell-Cycle Inhibition and Up regulation of Immune Response with Abemaciclib and Anastrozole in neoMONARCH, Phase II Neoadjuvant Study in HR+/HER2- Breast Cancer. Clin Cancer Res. (2020) 26:566–80. doi: 10.1158/1078-0432.CCR-19-1425, PMID: 31615937 PMC7498177

[21] Kuerer HM Buzdar AU Singletary SE . Biologic basis and evolving role of aromatase inhibitors in the management of invasive carcinoma of the breast. J SurgOncol. (2001) 77:139–47. doi: 10.1002/jso.1085, PMID: 11398169

[22] Early Breast CancerTrialists’ Collaborative Group (EBCTCG) . Relevance of breast cancer hormone receptors and other factors to the efficacy of adjuvant tamoxifen: patient-level meta-analysis of randomized trials. Lancet. (2011) 378:771–84. doi: 10.1016/S0140-6736(11)60993-8, PMID: 21802721 PMC3163848

[23] Gray RG Rea D Handley K . aTTom: Long-term effects of continuing adjuvant tamoxifen to 10 years versus stopping at 5 years in 6,953 women with early breast cancer. J ClinicalOncology. (2013) 31:5–5. doi: 10.1200/jco.2013.31.18_suppl.5

[24] Pagani O Regan MM Walley BA Fleming GF Colleoni M Láng I . Adjuvant exemestane with ovarian suppression in premenopausal breast cancer. N Engl J Med. (2014) 371:107–18. doi: 10.1056/NEJMoa1404037, PMID: 24881463 PMC4175521

[25] Francis PA Pagani O Fleming GF Walley BA Colleoni M Láng I . Tailoring adjuvant endocrine therapy for premenopausal breast cancer. N Engl J Med. (2018) 379:122–37. doi: 10.1056/NEJMoa1803164, PMID: 29863451 PMC6193457

[26] Baum M Budzar AU Cuzick J Forbes J Houghton JH Klijn JG . Anastrozole alone or in combination with tamoxifen versus tamoxifen alone for adjuvant treatment of postmenopausal women with early breast cancer: first results of the ATAC randomised trial. Lancet. (2002) 359:2131–9. doi: 10.1016/s0140-6736(02)09088-8, PMID: 12090977

[27] Breast International Group (BIG) 1–98 Collaborative Group Thurlimann B Keshaviah A AS C . A comparison of letrozole and tamoxifen in postmenopausal women with early breast cancer. N Engl J Med. (2005) 353:2747–57. doi: 10.1056/NEJMoa052258, PMID: 16382061

[28] Arimidex, Tamoxifen, Alone or in Combination (ATAC) Trialists’ Group Forbes JF Cuzick J Buzdar A . Effect of anastrozole and tamoxifen as adjuvant treatment for early-stage breast cancer: 100-month analysis of the ATAC trial. Lancet Oncol. (2008) 9:45–5. doi: 10.1016/S1470-2045(07)70385-6, PMID: 18083636

[29] NCCN . NCCN clinical practice guideline in oncology (NCCN guidelines^®^). In: Breast cancer, version 1 2022 Fort Washington, PA: National Comprehensive. Available online at: www.nccn.org/patients (Accessed 2022).

[30] Early breast cancer trialists’ Collaborative group (EBCTCG). Lancet. (2005) 365:1687–717. 10.1016/S0140-6736(14)62240-6 25457913

[31] Harbeck N Rastogi P Martin M Tolaney SM Shao ZM Fasching PA . Adjuvant Abemaciclib combined with endocrine therapy for high-risk early breast cancer: updated efficacy and Ki-67 analysis from the monarch study. AnnOncol. (2021) 32:1571–81. doi: 10.1016/j.annonc.2021.09.015, PMID: 34656740

[32] Johnston SRD Harbeck N Hegg R Toi M Martin M Shao ZM monarchE Committee Members and Investigators . Abemaciclib combined with endocrine therapy for the adjuvant treatment of HR+, HER2-, node-positive, high-risk, early breast cancer (monarchE). J. Clin Oncol. (2020) 38:3987–98. doi: 10.1200/JCO.20.02514, PMID: 32954927 PMC7768339

[33] Tolaney S Blancas I Im Y-H Rastogi P . (2021). Patients’ quality of life and side-effect perceptions in monarchE, a study of abemaciclib plus endocrine therapy in adjuvant treatment of HR+, HER2–, node-positive, high-risk, early breast cancer. Poster presentation at: The 17th St, in: Gallen International Breast Cancer Conference, , March 17-21, 2021. p. P008.

[34] Harbeck N Johnston S Fasching PA . High Ki-67 as a biomarker for identifying patients with high risk early breast cancer treated in monarchE. In: Poster presented at: San Antonio Breast Cancer Symposium. San Antonio, Texas, United States (2020).

[35] Rugo HS O’Shaughnessy J Boyle F Toi M Broom R Blancas I . Adjuvant abemaciclib combined with endocrine therapy for high risk early breast cancer: safety and patient-reported outcomes from the monarch E study S0923-7534(22)00383-0 doi: 10.1016/j.annonc.2022.03.00. 35337972

[36] Hudis CA Barlow WE Costantino JP Gray RJ Pritchard KI Chapman J-AW . Proposal for standardized definitions for efficacy end points in adjuvant breast cancer trials: the STEEP system. J ClinOncol. (2007) 25:2127–32. doi: 10.1200/JCO.2006.10.3523, PMID: 17513820

[37] Sledge GW Jr Toi M Neven P Sohn J Inoue K Pivot X . MONARCH 2: abemaciclib in combination with fulvestrant in women with HR+/HER2- advanced breast cancer who had progressed while receiving endocrine therapy. J Clin Oncol. (2017) 35:2875–84. doi: 10.1200/JCO.2017.73.7585, PMID: 28580882

[38] Goetz MP Toi M Campone M Sohn J Paluch-Shimon S Huober J . MONARCH 3: abemaciclib as initial therapy for advanced breast cancer. J Clin Oncol. (2017) 35:3638–46. doi: 10.1200/JCO.2017.75.6155, PMID: 28968163

[39] Sledge GW Jr Toi M Neven P Sohn J Inoue K Pivot X . The effect of abemaciclib plus fulvestrant on overall survival in hormone receptor-positive, ERBB2-negative breast cancer that progressed on endocrine therapy-MONARCH 2: A randomized clinical trial. JAMA Oncol. (2020) 6:116–24. doi: 10.1001/jamaoncol.2019.4782, PMID: 31563959 PMC6777264

[40] Johnston S O’Shaughnessy J Martin M Huober J Toi M Sohn J . Abemaciclib as initial therapy for advanced breast cancer: MONARCH 3 updated results in prognostic subgroups. NPJ Breast Cancer. (2021) 7:80. doi: 10.1038/s41523-021-00289-7, PMID: 34158513 PMC8219718

[41] Johnston S Martin M Di Leo A Im S Awada A Forrester T . MONARCH 3 final PFS: a randomized study of Abemaciclib as initial therapy for advanced breast cancer. NPJ Breast Cancer. (2019) 5:5. doi: 10.1038/s41523-018-0097-z, PMID: 30675515 PMC6336880

[42] Johnston S Toi M O’Shaughnessy J Rastogi P Campone M Neven P . Lancet Oncol. (2022). doi: 10.1016/S1470-2045(22)00694-5, PMID: 36493792 PMC11200328

[43] Hudis C Barlow W Costantino J Gray R Pritchard K Chapman J . S’Antonio breast cancer. J ClinOncol. (2007) 25:2127–32. doi: 10.1200/JCO.2006.10.3523, PMID: 17513820

[44] Toi M Boyle F Im YH Reinisch M Molthrop D Jiang Z . Adjuvant Abemaciclib Combined with Endocrine Therapy: Efficacy Results in monarchE Cohort 1. Oncologist. (2023) 28(1):e77–e81. doi: 10.1093/oncolo/oyac234, PMID: 36342342 PMC9847542

[45] Rugo HS . The importance of distant metastases in hormone-sensitive breast cancer. Breast. (2008) 17 Suppl 1:S3–8. doi: 10.1016/S0960-9776(08)70002-X, PMID: 18279764

[46] Gerber B Freund M Reimer T . Recurrent breast cancer: treatment strategies for maintaining and prolonging good quality of life. Dtsch Arztebl Int. (2010) 107:85–91. doi: 10.3238/arztebl.2010.0085, PMID: 20204119 PMC2832109

[47] Helwick C . Update from monarchE: benefit of abemaciclib increases over time in early-stage breast cancer. In: Supplement: breast cancer almanac (2023).

[48] Johnston SRD Toi M O’Shaughnessy J Rastogi P Campone M Neven P . Abemaciclib plus endocrine therapy for hormone receptor-positive, HER2-negative, node-positive, high-risk early breast cancer (monarchE): results from a preplanned interim analysis of a randomised, open-label, phase 3 trial. (2023). 24(1):77–90. doi: 10.1016/S1470-2045(22)00694-5, PMID: 36493792 PMC11200328

[49] Navarro-Yepes J Kettner NM Rao X Bishop CS Bui TN Wingate HF . Abemaciclib is effective in palbociclib-resistant hormone receptor–positive metastatic breast cancers. Cancer Res. (2023) 83:3264–83. doi: 10.1158/0008-5472.CAN-23-0705, PMID: 37384539 PMC10592446

[50] Holleczek B Jansen L Brenner H . Breast cancer survival in Germany: a population-based high resolution study from Saarland. PLoSOne. (2013) 8:e70680. doi: 10.1371/journal.pone.0070680, PMID: 23936237 PMC3729832

[51] Early Breast Cancer Trialists’ Collaborative Group (EBCTCG) . Aromatase inhibitors versus tamoxifen in early breast cancer: patient-level meta-analysis of the randomised trials. Lancet. (2015) 386:1341–52. doi: 10.1016/S0140-6736(15)61074-1, PMID: 26211827

[52] Dowsett M Turner N . Estimating risk of recurrence for early breast cancer: integrating clinical and genomic risk. J Clin Oncol. (2019) 37:689–92. doi: 10.1200/JCO.18.01412, PMID: 30742561

[53] Mennini FS Trabucco Aurilio M Gazzillo S Nardone C Sciattella P Marcellusi A An analysis of the social and economic costs of breast cancer in Italy. Int J Environ Res Public Health. (2021). 10.3390/ijerph18179005 PMC8431604 34501588

[54] Piccinni C Dondi L Ronconi G Calabria S Pedrini A Esposito I R+/HER2- metastatic breast cancer: epidemiology, prescription patterns, healthcare resource utilization and costs from a large italian real-world database. Clin Drug Investig. (2019) 39:945–51. doi: 10.1007/s40261-019-00822-4, PMID: 31347036

[55] Winer E . Adjuvant endocrine therapy for pre- and postmenopausal women. Breast. (2021) 56:S4–5. doi: 10.1016/S0960-9776(21)00053-9

[56] NICE . Abemaciclib with endocrine therapy for treating high-risk early breast cancer. In: Technology appraisal guidance \[TA761]. National Institute for Health and Care Excellence, London (2022).

[57] Jongbloed EM Blommestein HM van Schoubroeck HM Martens JWM Wilting SM Uyl-de Groot CA . Cost effectiveness of Abemaciclib as adjuvant therapy for HR+, HER2– early breast cancer. J Clin Oncol. (2023) 41:507. doi: 10.2147/BCTT.S387375, PMID: 36814469 PMC9940501

