# Correction: Abemaciclib early access - an Italian experience

**DOI:** 10.3389/fonc.2025.1734231

**Published:** 2025-11-11

**Authors:** Concetta Calabrò, Eleonora Cannella, Patrizia Nardulli

**Affiliations:** 1 Struttura Complessa di Farmacia IRCCS Istituto Tumori “Giovanni Paolo II”, Bari, Italy; 2 Pharmacy Department, Provincial Health Authority of Syracuse, Syracuse, Italy

**Keywords:** early access, cost - saving, breast cancer, C(nn) category, sustainability

Affiliation “Struttura Complessa di Farmacia IRCCS Istituto Tumori “Giovanni Paolo II” Bari, Italy” was omitted for authors “Calabrò Concetta” and “Patrizia Nardulli”.

The **Conflict of interest** statement has been correct to “The research was conducted in the absence of any commercial or financial relationships that could be construed as a potential conflict of interest.”

